# QTL Mapping Combined With Bulked Segregant Analysis Identify SNP Markers Linked to Leaf Shape Traits in *Pisum sativum* Using SLAF Sequencing

**DOI:** 10.3389/fgene.2018.00615

**Published:** 2018-12-05

**Authors:** Yuanting Zheng, Fei Xu, Qikai Li, Gangjun Wang, Na Liu, Yaming Gong, Lulu Li, Zhong-Hua Chen, Shengchun Xu

**Affiliations:** ^1^Central Laboratory of Zhejiang Academy of Agricultural Sciences, Hangzhou, China; ^2^College of Agriculture & Biotechnology, Zhejiang University, Hangzhou, China; ^3^School of Science and Health, Hawkesbury Institute for the Environment, Western Sydney University, Penrith, NSW, Australia

**Keywords:** bulked segregant analysis, high-density genetic map, leaf shape, *Pisum sativum*, specific locus amplified fragment sequencing

## Abstract

Leaf shape is an important trait that influences the utilization rate of light, and affects quality and yield of pea (*Pisum sativum*). In the present study, a joint method of high-density genetic mapping using specific locus amplified fragment sequencing (SLAF-seq) and bulked segregant analysis (BSA) was applied to rapidly detect loci with leaf shape traits. A total of 7,146 polymorphic SLAFs containing 12,213 SNP markers were employed to construct a high-density genetic map for pea. We conducted quantitative trait locus (QTL) mapping on an F_2_ population to identify QTLs associated with leaf shape traits. Moreover, SLAF-BSA was conducted on the same F_2_ population to identify the single nucleotide polymorphism (SNP) markers linked to leaf shape in pea. Two QTLs (qLeaf_or-1, qLeaf_or-2) were mapped on linkage group 7 (LG7) for pea leaf shape. Through alignment of SLAF markers with *Cicer arietinum, Medicago truncatula*, and *Glycine max*, the pea LGs were assigned to their corresponding homologous chromosomal groups. The comparative genetic analysis showed that pea is more closely related to *M. truncatula*. Based on the sequencing results of two pools with different leaf shape, 179 associated markers were obtained after association analysis. The joint analysis of SLAF-seq and BSA showed that the QTLs obtained from mapping on a high-density genetic map are convincing due to the closely associated map region with the BSA results, which provided more potential markers related to leaf shape. Thus, the identified QTLs could be used in marker-assisted selection for pea breeding in the future. Our study revealed that joint analysis of QTL mapping on a high-density genetic map and BSA-seq is a cost-effective and accurate method to reveal genetic architecture of target traits in plant species without a reference genome.

## Introduction

**FIGURE 1:**
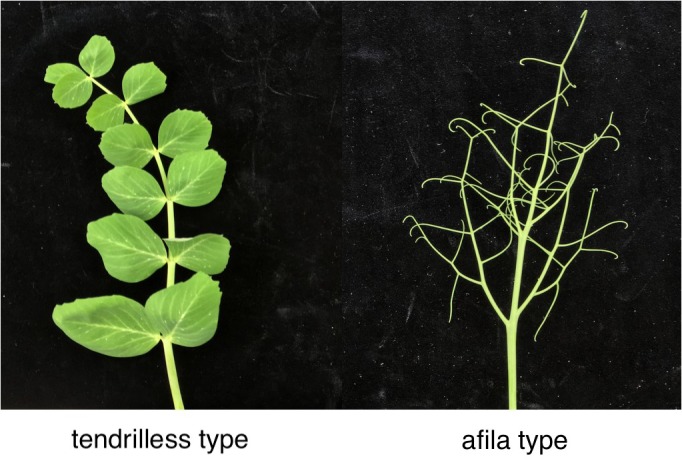
Different leaf shape of pea. The PS002 has *afila* leaf type and the PS047 has *tendrilless* leaf type.

Pea (*Pisum sativum* L.) is the fourth largest edible beans in the world (Gong et al., 2010). In the past few decades, pea has not only been planted as a highly nutritious crop, but also as an ideal plant species for studying genetic variation (Wang et al., 2008; Dahl et al., 2012). Leaf shape, including *tendrilless* and *afila* type (Figure 1), affects the utilization of solar radiation, the quality and yield of crops (Prajapati and Kumar, 2001; DeMason et al., 2013). Understanding the genetic architecture underlying leaf shape traits is therefore important for pea breeding programs.

Leaf shape is a quantitative trait controlled by multiple genes (Villani and DeMason, 1997, 1999, 2000). Previously, several genes (e.g., *Af, Mpf, Uni*, and *Tl*) related to leaf shape traits have been identified in pea (Villani and DeMason, 1999; Prajapati and Kumar, 2002; Mishra et al., 2009). There were complex regulatory relationships among these genes (Prajapati and Kumar, 2002; Kumar et al., 2004). Moreover, researchers also found that endogenous auxin and gibberellin pathways are involved in the formation of leaf shape in pea (DeMason and Chawla, 2004; DeMason et al., 2013). However, the regulatory network of leaf shape genes in pea is still unclear, which hinders the development of relevant molecular markers for breeding.

The development of massive number of molecular markers is the basis of molecular assisted selection (MAS), which is highly useful for plant breeding through the association between phenotypes and molecular markers. A huge number of molecular markers have been identified for constructing genetic map of pea, such as restriction fragment length polymorphism (RFLP), amplified fragment length polymorphism (AFLP), sequence-tagged sites (STS), cleaved amplified polymorphic sequences (CAP), inter simple sequence repeat (ISSR), and expressed sequence tag-simple sequence repeat (EST-SSR) (Weeden and Wolko, 1990; Timmerman-Vaughan et al., 1996; Gilpin et al., 1997; Irzykowska, 2001; Xu et al., 2012). QTL mapping is an efficient way to identify candidate genes related with phenotypes in pea. For example, a number of pea QTLs for grain yields, protein content, and plant height have been detected previously (Tar’An et al., 2003, 2004; Irzykowska and Wolko, 2004). However, fine mapping for the pea traits is difficult due to the low densities of the genetic markers (Jha et al., 2017). With the development of genotyping-by-sequencing (GBS), the resolution of QTL mapping has been improved significantly by the increasing numbers of markers. Specific locus amplified fragment sequencing (SLAF-Seq) has been used as a novel method for large-scale *de novo* SNP discovery (Sun et al., 2013). For instance, using SLAF-Seq, a high-density genetic map was constructed for white jute to identify QTLs for plant height (Tao et al., 2017). Bulked segregant analysis (BSA) is another way to discover genetic determinants underling phenotypic variants (Asnaghi et al., 2004). SLAF-BSA was used to identify SNP markers linked to dwarf traits in Lagerstroemia (Ye et al., 2016). Moreover, combining QTL mapping with high-density genetic map and bulked segregant RNA sequencing (SLAF-BSR) on F_2_ population, the genetic architecture of the maize kernel row number was revealed (Liu et al., 2016).

In this study, we applied a joint method of SLAF-seq and BSA to identify loci with leaf shape traits in pea. An F_2_ population was constructed from a cross between two lines with *tendrilless* and *afila* types. We constructed a high-density genetic map and QTL mapping for leaf traits using SLAF-Seq on this F_2_ population. Moreover, SLAF-BSA was conducted on the same F_2_ population to identify the SNP markers linked to leaf shape trait. Our result shows that the joint method was efficient and low-cost to mapping QTLs in pea. The investigation into the molecular genetics underlying pea leaf traits is not only important for understanding the genetic mechanisms for leaf shape development, but also vital for improving the production and quality of pea in the future.

## Materials and Methods

### Plant Materials and Phenotype Data Collection

A cross was made between two pea genotypes with different leaf shape, PS002 (*afila* leaf type) and PS047 (*tendrilless* leaf type) (Figure 1), to develop a F_2_ population at Zhejiang Academy of Agricultural Science (ZAAS), China. The phenotypic trait of the F_2_ population was collected at field station of ZAAS in 2017. The different leaf shapes (Figure 1) were evaluated for each individual according with previous study (DeMason et al., 2013).

### DNA Extraction

The genomic DNA of the 145 F_2_ plants and two parents were extracted from leaves following the protocol of the Plant Genomic DNA Kit (TIANGEN, Bejing, China). DNA concentration and quality were measured with a ND-1000 spectrophotometer.

### SLAF Library Construction and High-Throughput Sequencing

The SLAF-seq was utilized in this study (Sun et al., 2013). For the F_2_ population, the genomic DNA was digested using RsaI and HaeIII (New England Biolabs, NEB, United States). A single nucleotide (A) overhang was added subsequently to the digested fragments using Klenow Fragment (3′ì→ 5′ì exo–) (NEB) and dATP at 37°C. T4 DNA ligase was used to ligate the duplex tag-labeled sequencing adapters (PAGE-purified, Life Technologies, United States) to the A-tailed fragments. PCR was used with diluted restriction-ligation DNA samples, dNTP, Q5^®^ High-Fidelity DNA Polymerase and PCR primers (Forward primer: 5′-AATGATACGGCGACCACCGA-3′, reverse primer: 5′-CAAGCAGAAGACGGCATACG-3′; PAGE-purified, Life Technologies, United States). PCR products were then purified using Agencourt AMPure XP beads (Beckman Coulter, High Wycombe, United Kingdom) and pooled. Pooled samples were separated by 2% agarose gel electrophoresis. Fragments ranging from 264 to 314 base pairs (with indexes and adaptors) in size were excised and purified using a QIAquick gel extraction kit (Qiagen, Hilden, Germany). Gel-purified products were then diluted. Pair-end sequencing (125 bp) was utilized on an Illumina HiSeq 2500 system (Illumina, Inc; San Diego, CA, United States) following the manufacturer’s recommendations.

### SLAF-seq Data Analysis and Genotyping

The sequencing data have been deposited into the NCBI/GEO database with accession number PRJNA494031. The identification and genotyping of SLAF markers were conducted using procedures described by Sun et al. (2013). In brief, low-quality reads were filtered out and then raw reads were sorted to each progeny according to duplex barcode sequences. After the barcodes and the terminal 5-bp positions were trimmed from each high-quality read. Clean reads were clustered by similarity above 90%. Sequences clustered together were defined as one SLAF locus (Zhang J. et al., 2015b). Single nucleotide polymorphism (SNP) loci of each SLAF locus were then identified between parents, and SLAFs with more than three SNPs were filtered out. Alleles of each SLAF locus were then defined according to parental reads with sequence depth >35.87-fold, while for each offspring the reads with sequence depth >10.40-fold were used to define alleles. Then repetitive SLAFs were defined and discarded subsequently. Only SLAFs with 2–4 alleles were identified as polymorphic and considered potential markers. All polymorphism SLAFs loci were genotyped in the parental and offspring SNP loci. The marker code of the polymorphic SLAFs was analyzed according to the population type F_2_, which consist of one segregation type (aa × bb).

Genotype scoring was then conducted using a Bayesian approach to reassurance of the genotyping quality (Sun et al., 2013). *A posteriori* conditional probability was calculated using the coverage of each allele and the number of single nucleotide polymorphism. Then, genotyping quality score translated from the probability was used to select qualified markers for subsequent analysis. Low-quality markers were counted and the worst marker or individual were deleted during the dynamic process (Sun et al., 2013). High-quality SLAF markers for the genetic mapping were filtered according to the following criteria: (1) average sequence depths >10-fold in the parents, (2) markers with more than 70% missing data were filtered, and (3) the chi-square test was performed to examine the segregation distortion (Sun et al., 2013). The filter criteria for high quality markers are (1) The sequence depths of SLAFs in PS002 and PS047 were larger than 10-fold, (2) The number of SNP was greater than 5, (3) Elimination of polymorphic tags with parental heterozygosis, (4) The screened genotype should cover at least 70% of all progeny markers, and (5) Segregation distortion mark filtering.

### Linkage Map Construct and QTL Mapping

Marker loci were partitioned primarily into linkage groups (LGs) by the modified logarithm of odds (MLOD) scores >5. The HighMap strategy was performed to order the SLAF markers and correct genotyping errors within LGs (Liu et al., 2014). Firstly, recombinant frequencies and LOD scores were calculated by two-point analysis, which were applied to infer linkage phases. Then, enhanced gibbs sampling, spatial sampling and simulated annealing algorithms were combined to conduct an iterative process of marker ordering (Jansen et al., 2001; Van Ooijen, 2011). Simulated annealing was applied to searching for the best map order. Summation of adjacent recombination fractions was calculated as illustrated by Liu et al. (2014). Blocked Gibbs sampling was employed to estimate multipoint recombination frequencies of the parents after the optimal map order of sample markers were obtained (Liu et al., 2014). The error correction strategy of SMOOTH was then conducted according to parental contribution of genotypes (Os et al., 2005) and a k-nearest neighbor algorithm was applied to impute missing genotypes (Huang et al., 2012). Skewed markers were then added into this map by applying a multipoint method of maximum likelihood. Map distances were estimated using the Kosambi (1943) mapping function. The R/qtl package was used for QTL mapping for leaf shape. The IM method of the R/qtl package was applied for qualitative traits mapping (Broman and Sen, 2009). The threshold of the logarithm of odds (LOD) scores was determined using 1,000 permutations.

### SLAF-BSA

Based on leaf shape of pea, 30 individual lines with tendrils leaf type and 30 individual lines with tendrilless leaf type from the F_2_ population were selected and grouped as two bulks, namely A pool (A-pool) and B pool (B-pool), for the BSA of the year 2017. DNA extraction and SLAF library construction were the same as we talked before in method 4. The average sequencing depths were more than 56.21-fold in the parents and 62.31-fold in the progeny pools averagely. Sequence similarity was detected by BLAT (Kent, 2002), and sequences with over 90% identity were defined as a SLAF locus. In each of the SLAF loci, we examined the polymorphism locus between the parents. Then, all of the polymorphic SLAFs were genotyped in the progeny as well as in any offspring containing more than 80% of the SLAFs in the parents. Potential SLAFs with one genotype originating from M and the other from P were identified as markers. Then the Δ (SNP-index) was used in association analysis for finding significant differences in genotype frequencies between pools (Abe et al., 2012; Takagi et al., 2013). The method to calculate the SNP-index is as follows: Maa indicated the depth of aa population originated from M; Paa indicated the depth of AA population originated from P; Mab indicated the depth of ab population originates from M; while Pab indicated depth of ab population originates from P.

$$\begin{array}{l} \text{SNP}-\text{index}(ab)=\text{Mab}/(\text{Pab}+\mathrm{Mab});\\ \text{SNP}-\text{index}(\mathrm{aa})=\text{Maa}/(\mathrm{Paa}+\mathrm{Maa});\\ \Delta (\mathrm{SNP}-\mathrm{index})=\mathrm{SNP}-\mathrm{index}(\mathrm{aa})-\mathrm{SNP}-\mathrm{index}(\mathrm{ab}); \end{array}$$

In the method of determining the Δ(SNP-index) threshold of non-reference genome, Δ(SNP-index) greater than 0.99 Marker was selected as the threshold of significant association with traits, while the marker larger than the threshold was the marker of significant association with traits.

### Analysis of Co-linearity and SLAF Markers Annotation

The sequences of the SLAF markers included in the linkage map were aligned to the physical sequences of barrel medic (*Medicago Truncatula*)^1^, chickpea (*Cicer arietinum*)^2^, and soybean (*Glycine max*)^3^. The tool used in the co-linearity study is mummer. The SLAF markers were annotated against the KOG/COG, GO, KEGG, Pfam, Swissprot, TrEMBL, eggNOG, Nr, and Nt database.

## Results

### Analysis of SLAF-seq Data and SLAF-Markers

**FIGURE 2:**
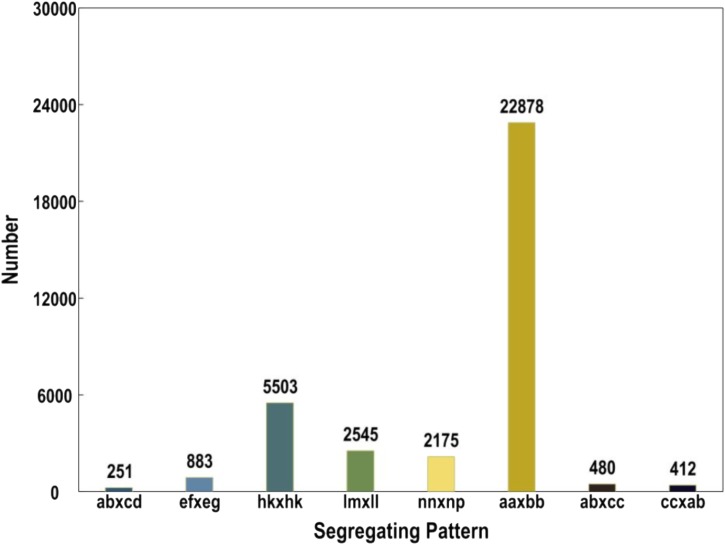
Genotype distribution of Specific locus amplified fragment (SLAF) markers in pea. The *x*-axis indicates eight segregation patterns of polymorphic SLAF markers and *y*-axis indicates the number of markers.

A total of 669,769,663 reads were generated from the SLAF-seq. The average percentage of Q30 bases was 92.40% and the average guanine-cytosine (GC) content was 40.71%. In our study, we use rice (*Oryza sativa* L. *Japonica*, genome size = 374.30M^4^) as a control to estimate the validity of library construction. We obtained 938,017 total reads in *O. sativa* with 91.14% Q30 bases and 45.72% GC content. Specifically, 19,507,395 and 18,318,183 reads were generated for PS047 and PS002, respectively, while 631,944,055 reads were obtained for 145 offspring. The Q30 for PS047, PS002 and their offspring were 92.37, 92.59, and 92.40%, respectively. GC content of PS047, PS002, and their offspring were very consistent at 40.93, 40.79, and 40.70%, respectively (Supplementary Table S1). Based on high quality pair-end reads and SLAF tag development process, the reads at the same location were used as a SLAF tag and a total of 358,458 SLAF tags were developed. The average sequencing depth of the SLAF tag in the parent lines was 35.87-fold and the average offspring sequencing depth was 10.40-fold (Supplementary Table S2). According to the differences between alleles and gene sequences, 3 types of polymorphisms: Polymorphic, Non-Polymorphic, and Repetitive, were obtained. In the 358,458 developed SLAF tags, polymorphic SLAF tags number is 49,632, the polymorphic ratio reached 13.85% (Table 1). Among the 49,632 polymorphic SLAFs, 22,878 SLAFs (46.1%) were successfully chosen for map construction according to the genotype of SLAF Markers (Figure 2).

**Table 1:** SLAF label type statistics.

Type	Polymorphic SLAF	Non-Polymorphic SLAF	Repetitive SLAF	Total SLAF
Number	49,632	307,118	1,708	358,458
Percentage	13.85%	85.68%	0.47%	100.00%

### The Construction and Characteristic of High-Density Genetic Map

**FIGURE 3:**
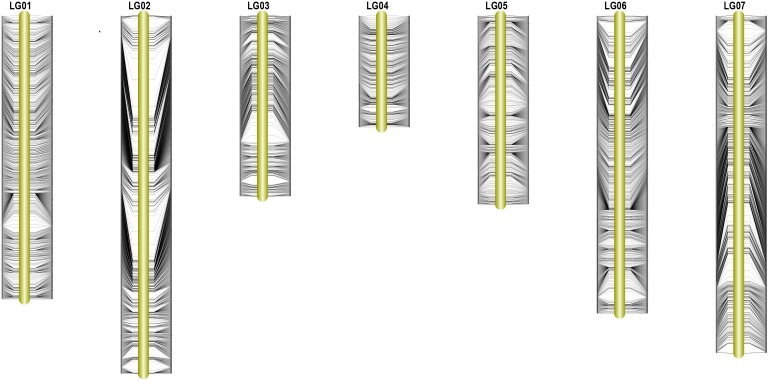
High-density linkage map of pea. Yellow columns represent the 7 LGs. SLAF markers are located according to genetic distance (cM).

In order to ensure the quality of the genetic map, 22,878 SLAFs were filtered further to 8,571 SLAFs. Then, the genotypes of 8571 SLAFs were analyzed to determine the order of the SLAF markers in 7 LGs by calculating the recombination rate between the two tags. Finally, a total of 7,146 SLAFs were on the map, account for 83.37%. Based on those analyses, we constructed a new high-density genetic map (Figure 3). The total genetic distance was 1120.81 cM. The largest LG was LG2, with 1456 SLAF markers and a length of 189.94 cM. The smallest LG was LG4, with 453 SLAF markers and a length of 141.57 cM (Table 2). In addition, a total of 12,213 SNP markers were on the map (Table 3). LG2 had the largest number (2,480) of SNP, while LG4 contained the lowest number (848) of SNP. The Tri/Trv result also showed difference between the seven LGs (Table 3).

**Table 2:** Description on basic characteristics of seven linkage groups.

Linkage group ID	Total marker	Total distance(cM)	Average distance(cM)	Gap < 5 cM	Max gap(cM)
LG1	1,153	146.34	0.13	100%	2.485
LG2	1,456	189.94	0.13	98.97%	14.145
LG3	734	148.05	0.2	99.18%	7.928
LG4	453	141.57	0.31	99.78%	5.22
LG5	767	146.32	0.19	99.74%	2.554
LG6	1,211	176.82	0.15	99.75%	5.889
LG7	1,372	171.77	0.13	99.78%	4.997

*Gap < 5 cM indicated the percentages of gaps in which the distance between adjacent markers was smaller than 5 cm*.

**Table 3:** SNP information for high density genetic map of pea.

Linkage group ID	SNP number	Tri/Trv
LG1	1,845	1,161/684
LG2	2,480	1,669/811
LG3	1,310	865/445
LG4	848	596/252
LG5	1,325	947/378
LG6	2,124	1,465/659
LG7	2,281	1,618/663
Total	12,213	8,321/3,892

*Tri, SNP transitions; Trv, SNP transversions*.

### Quality Evaluation of the Genetic Map

We constructed the haplotype and heat maps for quality-check of the high-density genetic map of pea. As shown in the haplotype maps of seven LGs, the source of the larger segment of each individual was consistent and most of the LGs were uniformly distributed, indicating the high quality of this genetic map (Supplementary Figure S1). The missing data for the seven LGs ranged from 0.06% (LG2) to 0.78% (LG4) (Supplementary Table S3). Based on the marker recombination relationship heat map (Supplementary Figure S2), the linkage relationship between adjacent markers on each linkage group was very strong, and the linkage relationship with the far-reaching markers was weak, indicating that the marking order is correct. We also constructed the integrity map for all individuals (Supplementary Figure S3), the average percentage of integrity is 97.54%, which guaranteed the accuracy of map genotyping.

The segregation distortion markers are likely to have an impact on QTL mapping, so the polymorphic markers with severe segregation (chi-square test *P* < 0.05) were filtered in our study (Zhang Y. et al., 2015c). We analyze the remaining segregation distortion markers (Supplementary Table S4) that were only distributed on the LG2 with a percentage of 1.03%. Furthermore, two segregation distortion region (SDR) were detected on LG2.

### Comparative Genomic Analysis

**FIGURE 4:**
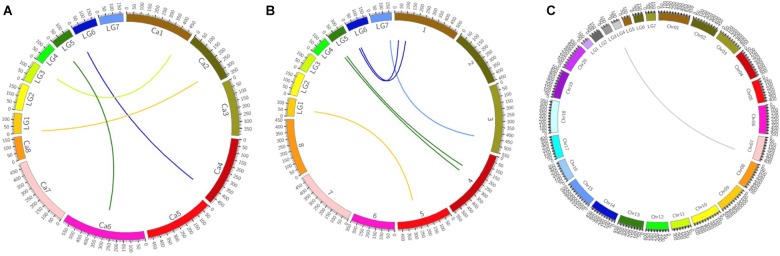
Circos plots of comparative genome between pea and three other species. **(A)** Relationships between pea and *Cicer arietinum* pea on genome level. **(B)** Relationships between pea and *Medicago truncatula* on genome level **(C)** Relationships between pea and *Glycine max* on genome level.

The total numbers of matching markers containing sequences between pea and each species were 1083 (0.3%) to *M. truncatula*; 870 (0.24%) to *C. arietinum*; 364 (0.1%) to *G. max* (Table 4). The 7,146 mapped SLAF markers were also assessed in the three species. The mapped marker followed a similar trend to that of all markers with 6 (0.08%) to *M. truncatula*; 4 (0.06%) to *C. arietinum*; 1 (0.01%) to *G. max*. Three Circos plots were used to show the relationships between pea and those species (Figure 4). These results indicate that pea is more closely related to *M. truncatula* than other tested species in this study.

**Table 4:** Comparative analyses of pea SLAF markers and genome of Medicago, Chickpea, and Soybean.

Species	All SLAF	Percentage	Mapped SLAF markers	Percentage
Pea	358458		7146	
Chickpea	870	0.24%	4	0.06%
Medicago	1083	0.30%	6	0.08%
Soybean	364	0.10%	1	0.01%

### QTLs Mapping of Leaf Shape

Two QTLs were mapped on LG7 for pea leaf shape and the LOD threshold values of the two QTLs were both 2.0 (Table 5). The QTL with the higher effect was qLeaf_or-2 with a LOD value of 2.47 and explained 7.16% of the phenotypic variance. Another QTL qLeaf_or-1 had a smaller effect with a LOD value of 2.30 and explained 6.56% of the phenotypic variance. Both qLeaf_or-1 and qLeaf_or-2 had an additive effect -0.03. The dominant effects for qLeaf_or-1 and qLeaf_or-2 were 0.16 and 0.17, respectively (Table 5). The genetic interval of qLeaf_or-2 was from 155.82 to 156.169, while qLeaf_or-1 was smaller (from 152.031 to 152.032). The QTL distribution results are presented in Supplementary Figure S4.

**Table 5:** Analysis of quantitative trait loci (QTLs) for leaf shape in pea.

QTLs	LOD threshold	Linkage	Start	End	Max LOD	PVE	ADD	DOM
qLeaf_or-1	2.0	7	152.031	152.032	2.30	6.56	-0.03	0.16
qLeaf_or-2	2.0	7	155.824	156.169	2.47	7.16	-0.03	0.17

*PVE indicates the phenotypic variance explained by individual QTL; ADD represents the additive effect of the QTL; DOM represents the dominant effect of the QTL*.

**FIGURE 5:**
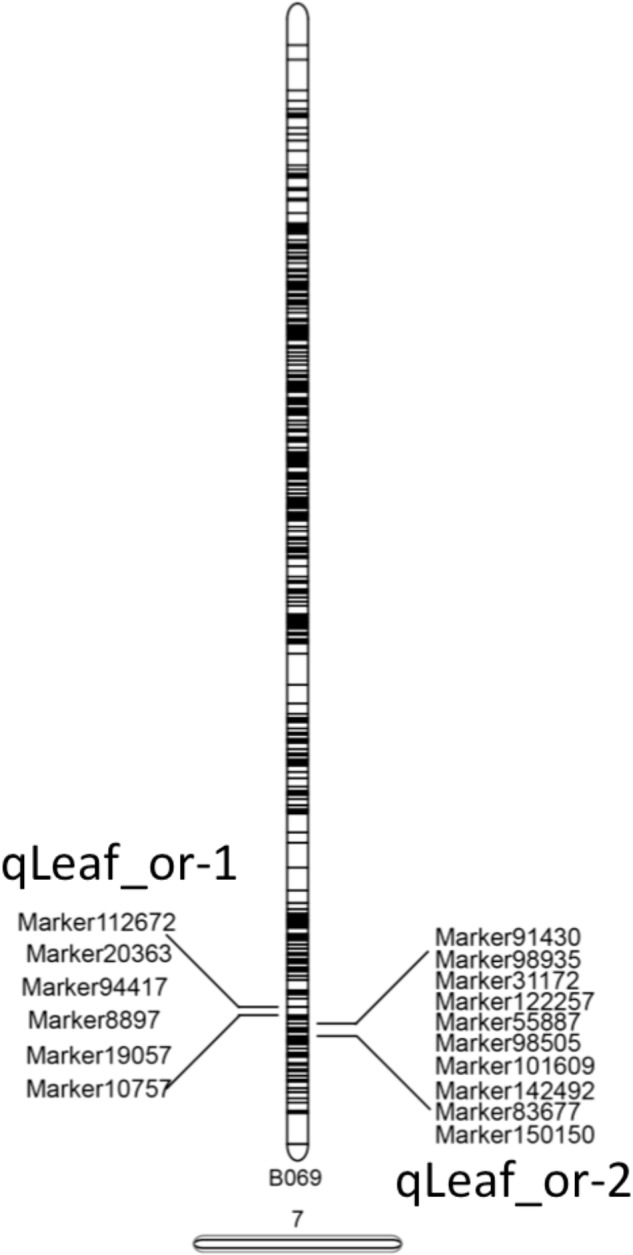
SLAF markers within two QTLs. Two QTLs were both in LG7, 6 SLAF markers were in qLeaf_or-1 and 10 SLAF markers were in qLeaf_or-2.

After the identification of two QTLs, we annotated the SLAF markers within the two QTLs to relative species database. Detail information about the two QTLs were described in Figure 5. There were sixteen SLAF markers within two QTLs. The blast result provided more information: (1) Ten SLAF markers had annotations in database with different molecular function. (2) Six SLAF markers annotation information were about polyprotein involved in nucleic acid binding, DNA metabolic process, hydrolase activity, catalytic activity, macromolecule metabolic process, recombination and repair hypothetical activity. (3) most of the SLAF markers are annotated in *Pisum sativum* and only one is annotated in *M. truncatula*. Further research needed to be conducted to explain the whole molecular network involved in pea leaf shape.

### BSA-seq for Pea Plant Morphology

A total of 67.85 M reads were obtained from BSA-seq. The average Q30 of sequencing was 93.64%, and the average GC content was 40.61%. Rice control sequencing was used to evaluate the accuracy of the experimental library, and the 0.97 M reads data was obtained (Supplementary Table S5). Then A total of 271,715 SLAF markers were developed. The average sequencing depth of SLAF tags from parents was 56.21-fold, and that of F_2_ mixed pools was 62.31-fold (Supplementary Table S6). Based on these data, a total of 271,715 polymorphic SLAF tags were developed, including 34,754 polymorphic SLAFs, 232,999 non-polymorphic SLAFs and 3,962 repetitive SLAFs (Table 6). A total of 17,860 polymorphic SLAF tags were screened for subsequent association analysis. Through the Δ SNP-index method, the 0.99 percentile was used as the threshold, and 179 markers with a significant association with the traits were obtained. The associated tag statistics are shown in Supplementary Table S7, where there were 320 SNP sites among the 179 associated markers.

**Table 6:** BSA SLAF Polymorphic Analysis.

Type	Polymorphic SLAF	Non-Polymorphic SLAF	Repetitive SLAF	Total SLAF
Number	34,754	232,999	3,962	271,715
Percentage **(%)**	12.79	85.75	1.46	100

### Combination Analysis of Genetic Map and BSA-seq

**FIGURE 6:**
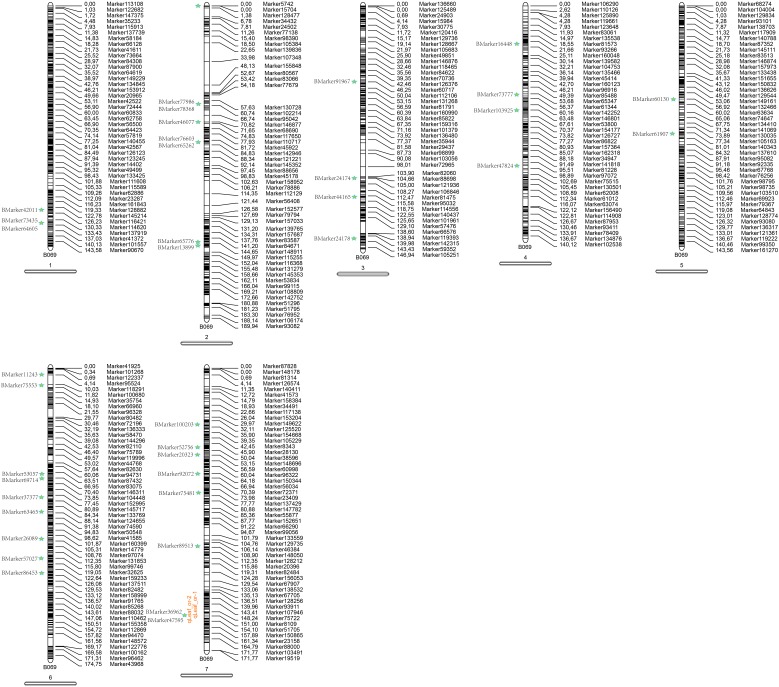
A joint map combing BSA and QTL. This map is based on the high-density genetic map. Green star represents the BSA markers. Orange locus represents the QTLs.

For further understanding of the genetic basis of pea leaf traits, a joint map containing the BSA markers and high-density map was constructed to obtain more comprehensive molecular marker information (Figure 6). A total of 37 BSA markers, which associated with leaf traits, were aligned on the genetic high-density map. The joint map showed that 20.67% of BSA markers could be drawn on the map. Two loci were around the result of QTL mapping on LG7, which are the most likely candidates for pea leaf shape. The LG6 contained the most BSA markers while the LG5 harbored the least BSA markers.

## Discussion

### SLAF-seq Is a Promising Tool for High-Density Genetic Map Construction With F_2_ Population of Pea

Quantitative trait locus mapping has been used to explore genetic architecture underlying quantitative traits in pea (Timmerman-Vaughan et al., 1996; Lejeune-Hénaut et al., 2008; Leonforte et al., 2013) for positional cloning with abundant genetic markers (Ellis and Poyser, 2002). The number of genetic markers on map in previous studied was lower than 500 (Ellis et al., 1992; Gilpin et al., 1997; Laucou et al., 1998; Weeden et al., 1998; Irzykowska, 2001; Ellis and Poyser, 2002). Recently, high throughput sequencing technology provided high density genetic map to get more accurate QTLs. However, the high cost prevents this method to be applied widely in plant breeding, especially for plants with large genomes. Fortunately, with the development of SLAF-seq, thousands of polymorphic markers could be generated to construct high density genetic maps with low-cost (Sun et al., 2013). Many studies have shown that SLAF-seq can increase the power of QTL mapping than traditional PCR-base method such as ice grass, soybeans, sweet cherry, cucumber, and sesame (Zhang Y. et al., 2013; Wei et al., 2014; Zhang J. et al., 2015a; Jin et al., 2016). In the present study, we obtained 49,632 polymorphic SLAFs for map construction and a high-density genetic map containing 12,213 SNP markers. Compared with previous pea genetic maps (Tar’An et al., 2003, 2004), our map has more genetic markers with a higher resolution.

In recent years, SLAF-seq has been applied in constructing high-density genetic map for crops, which have their own reference genomes (Goff et al., 2002; Dai et al., 2018; Wang et al., 2018). It is relatively easy for map construction and target gene identification for these crops. There were a few studies focusing on constructing genetic map by using SLAF-seq on non-reference genome plants, such as sesame (Zhang Y.X. et al., 2013) and orchardgrass (Zhao et al., 2016). *Pisum sativum* genome information has not yet been published, and its huge genome (4.45 Gbp) consists of high copy dispersed repeated sequences (Zhu et al., 2005). Therefore, it was important for a quality evaluation for the map constructed by SLAF-seq on pea. From the marker recombination relationship heat maps (Supplementary Figure S2), the linkage relationship between adjacent markers on each linkage group was strong and the linkage relationship with the far-reaching markers was weak. Combined with the low missing data rate information (Supplementary Table S4), the genetic map constructed in our work showed high quality. Our high density genetic map may be useful to locate novel QTLs for important traits, and serve as a reference for positioning sequence scaffolds on the physical map to contribute to the assembling of the pea genome in the future.

### Comprehensive Genetic Architecture Revealed by Joint Analysis of QTL Mapping and BSA-seq

Recently, high-density genetic map was used for QTL mapping of plant traits in many crops with reference genome and many important QTLs were utilized for gene annotation, cloning, and functional analysis (Li et al., 2017). Here, a low matching was found from comparative genome analysis among our sequence data with *M. truncatula, C. Arietinum*, and *G. max*. The results revealed that few reference genome information could be used in the present study. Generally, the QTL detection efficiency was limited in the F_2_ population because of recombination frequency in each individuals and the lack of genomic information (Liu et al., 2016; Tao et al., 2017). Surprisingly, only two QTLs were detected in this study, which leads to the speculation that the leaf shape traits might be controlled by multiple minor genes and involve complex molecular pathway. Thus, the strategies need to be improved in future work.

Bulked segregant analysis is another efficient method for the rapid identification of molecular markers linked to the target gene or genomic region in two bulks with clear phenotypic differentiation. In the past, the availability of DNA markers was the main factor limiting the effectiveness of BSA. Next-generation sequencing technologies provide a comprehensive and cost-effective means of BSA. BSA based on SLAF-seq circumvents the limitation of DNA marker availability and does not require complete genotyping. This strategy has been successfully applied to various plant species including pepper, Melon, cotton, cucumber and maize (Liu et al., 2016; Guo et al., 2017; Sheng et al., 2017; Li et al., 2018; Xu et al., 2018; Zhao et al., 2018). SLAF-seq-BSA showed more SLAF markers that are related to target traits than QTL mapping on genetic map.

In view of this reason, SLAF-seq-BSA were conducted as a supplementary means to detect genomic regions of leaf shape trait in the current study. We identified 179 markers associated with leaf shape traits. In order to comprehensively understand the results from both methods, we marked the coincident part of QTL mapping and BSA results on the same linkage map. It was clear that QTL mapping results are much less than the BSA (Figure 6). Two BSA markers were found to be close to the QTL region and the QTLs were validated by the BSA results. Moreover, it is unlikely to identify all the markers that contribute to leaf shape trait because of the lack of pea genome. Other markers that produced from BSA could be used as a supplement to mapping results, and could be potential candidates for future analysis. In maize, QTL mapping using a high-density genetic map and BSR-seq were combined to comprehensively illustrate the molecular markers of maize kernel row number (Liu et al., 2016). Our results indicated that QTL mapping and BSA methods complemented with each other. For plant species such as pea, which has no reference genome, the joint analysis of two methods will give a more convincing and reliable approach for genetic analysis and breeding programs. Our findings also provides a practical and useful way for the genetic mapping study of non-reference species.

## Conclusion

In summary, our study provided a high-density genetic map with SLAF-seq in pea. We used the joint genetic analysis of QTL mapping and BSA-seq to comprehensively study the genetic architecture underlying pea leaf shape traits. The QTL mapping results provided abundant information of pea leaf shape for future analysis and marker-assistant selection in pea breeding. The joint genetic analysis may be feasible and have broad applications in breeding programs for plant species without a reference genome.

## Availability of Data

The sequencing data have been deposited into the NCBI/GEO database with accession number PRJNA494031.

## Author Contributions

SX and Z-HC conceived and designed the experiments. YZ, QL, SX, GW, and NL performed the experiments. YZ, FX, LL, and SX analyzed the data. SX, NL, and YG contributed the reagents, materials, and analysis tools. YZ, FX, SX, and Z-HC wrote the paper. LL participated in the integration of genetic maps and QTL analysis process, and also participated as a major member in the revision of the “Discussion” section.

## Conflict of Interest Statement

The authors declare that the research was conducted in the absence of any commercial or financial relationships that could be construed as a potential conflict of interest.

## Footnotes

1 https://www.ncbi.nlm.nih.gov/genome/?term=txid3880[orgn]

2 https://www.ncbi.nlm.nih.gov/genome/?term=txid3827[orgn]

3 https://phytozome.jgi.doe.gov/pz/portal.html#!info?alias=Org_Gmax

4 http://rapdb.dna.affrc.go.jp/

## Supplementary Material

FIGURE S1 Haplotype maps of LG1-LG7.

FIGURE S2 Heat map for LG1-LG7.

FIGURE S3 Map integrity for all individuals. The *X*-axis represents the sample; the *Y*-axis represents completeness.

FIGURE S4 QTL distribution map. The *X*-axis is the ranking order of Marker in linkage group, the *Y*-axis is the LOD value, the gray line is the threshold line, and the area above the threshold line is the QTL area.

TABLE S1 Basic statistics of the SLAF-seq data.

TABLE S2 SLAF label statistics.

TABLE S3 The missing proportion of markers on map.

TABLE S4 Segregation Distortion Markers.

TABLE S5 BSA sequencing data statistics.

TABLE S6 BSA SLAF marker statistics.

TABLE S7 179 markers with a significant degree of association with the traits.
