# Supplementary figures and images for: QTL Mapping Combined With Bulked Segregant Analysis Identify SNP Markers Linked to Leaf Shape Traits in Pisum sativum Using SLAF Sequencing
Source: Front Genet. 2018 Dec 5;9:615. doi: 10.3389/fgene.2018.00615 (PMC6290080; doi:10.3389/fgene.2018.00615)

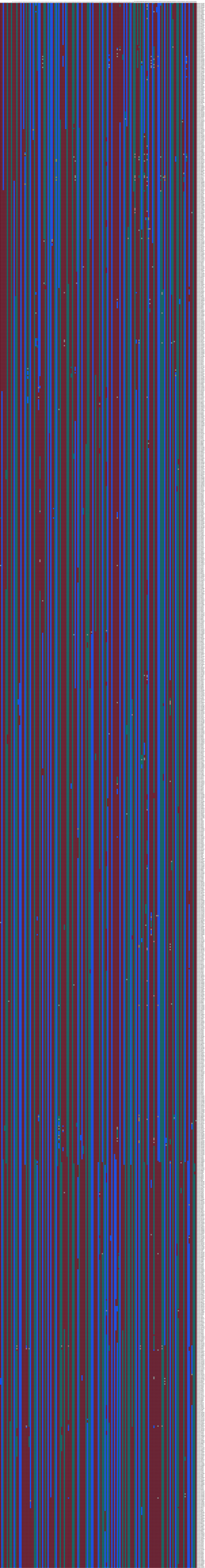

Supplement: FIGURE S1 — Haplotype maps of LG1-LG7. [file Data_Sheet_1.ZIP › Supplemental materials/Supplement Figure/Figure S1/LG1.haplo.jpg]

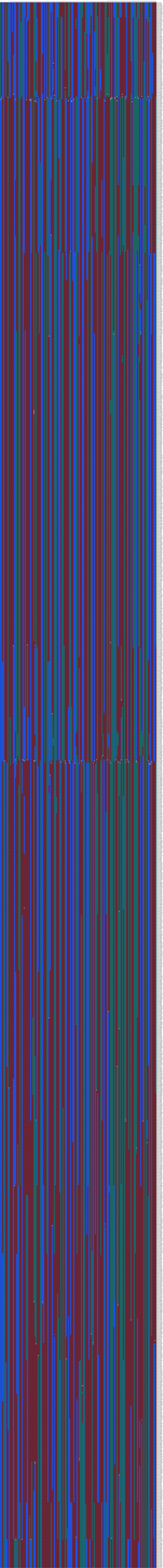

Supplement: FIGURE S1 — Haplotype maps of LG1-LG7. [file Data_Sheet_1.ZIP › Supplemental materials/Supplement Figure/Figure S1/LG2.haplo.jpg]

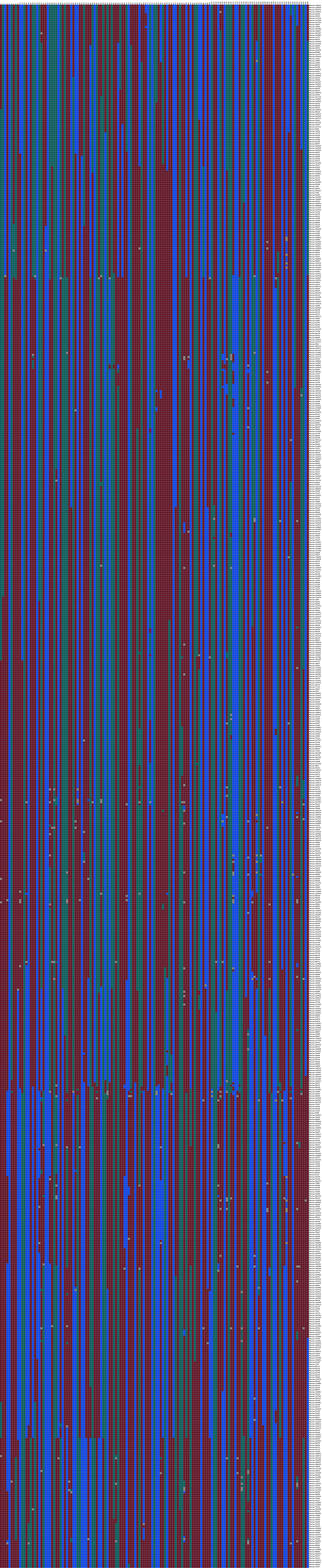

Supplement: FIGURE S1 — Haplotype maps of LG1-LG7. [file Data_Sheet_1.ZIP › Supplemental materials/Supplement Figure/Figure S1/LG3.haplo.jpg]

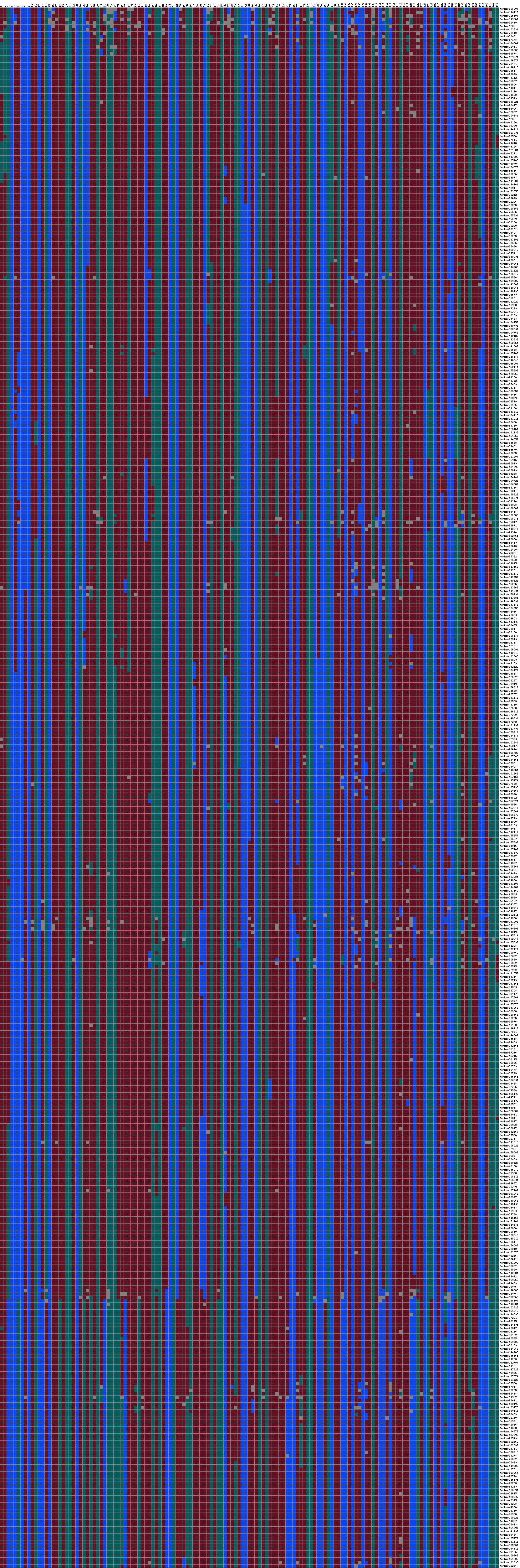

Supplement: FIGURE S1 — Haplotype maps of LG1-LG7. [file Data_Sheet_1.ZIP › Supplemental materials/Supplement Figure/Figure S1/LG4.haplo.jpg]

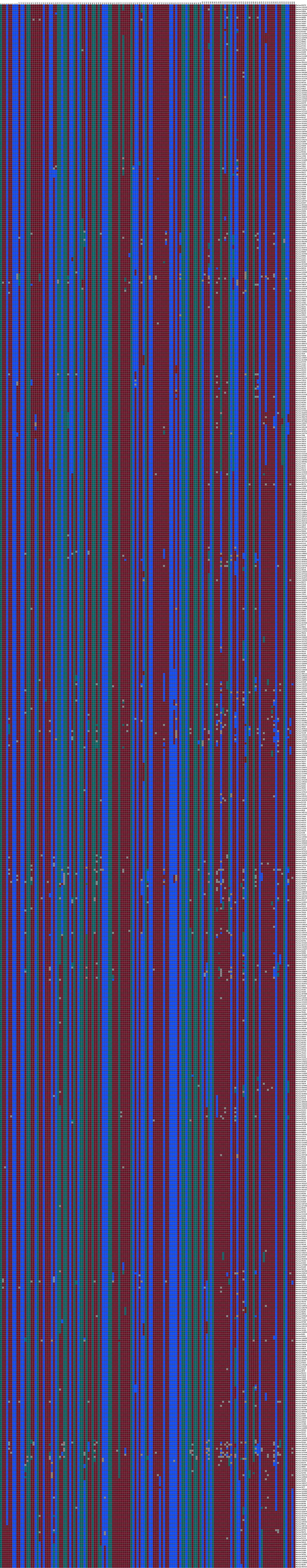

Supplement: FIGURE S1 — Haplotype maps of LG1-LG7. [file Data_Sheet_1.ZIP › Supplemental materials/Supplement Figure/Figure S1/LG5.haplo.jpg]

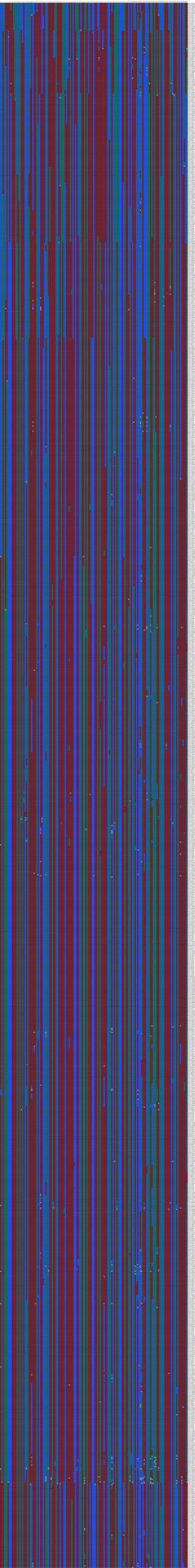

Supplement: FIGURE S1 — Haplotype maps of LG1-LG7. [file Data_Sheet_1.ZIP › Supplemental materials/Supplement Figure/Figure S1/LG6.haplo.jpg]

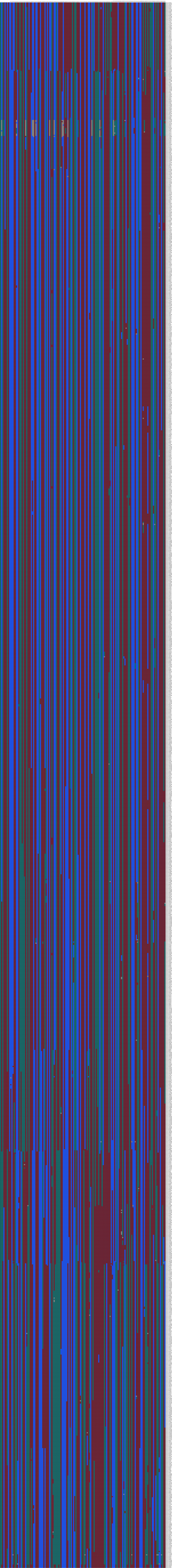

Supplement: FIGURE S1 — Haplotype maps of LG1-LG7. [file Data_Sheet_1.ZIP › Supplemental materials/Supplement Figure/Figure S1/LG7.haplo.jpg]

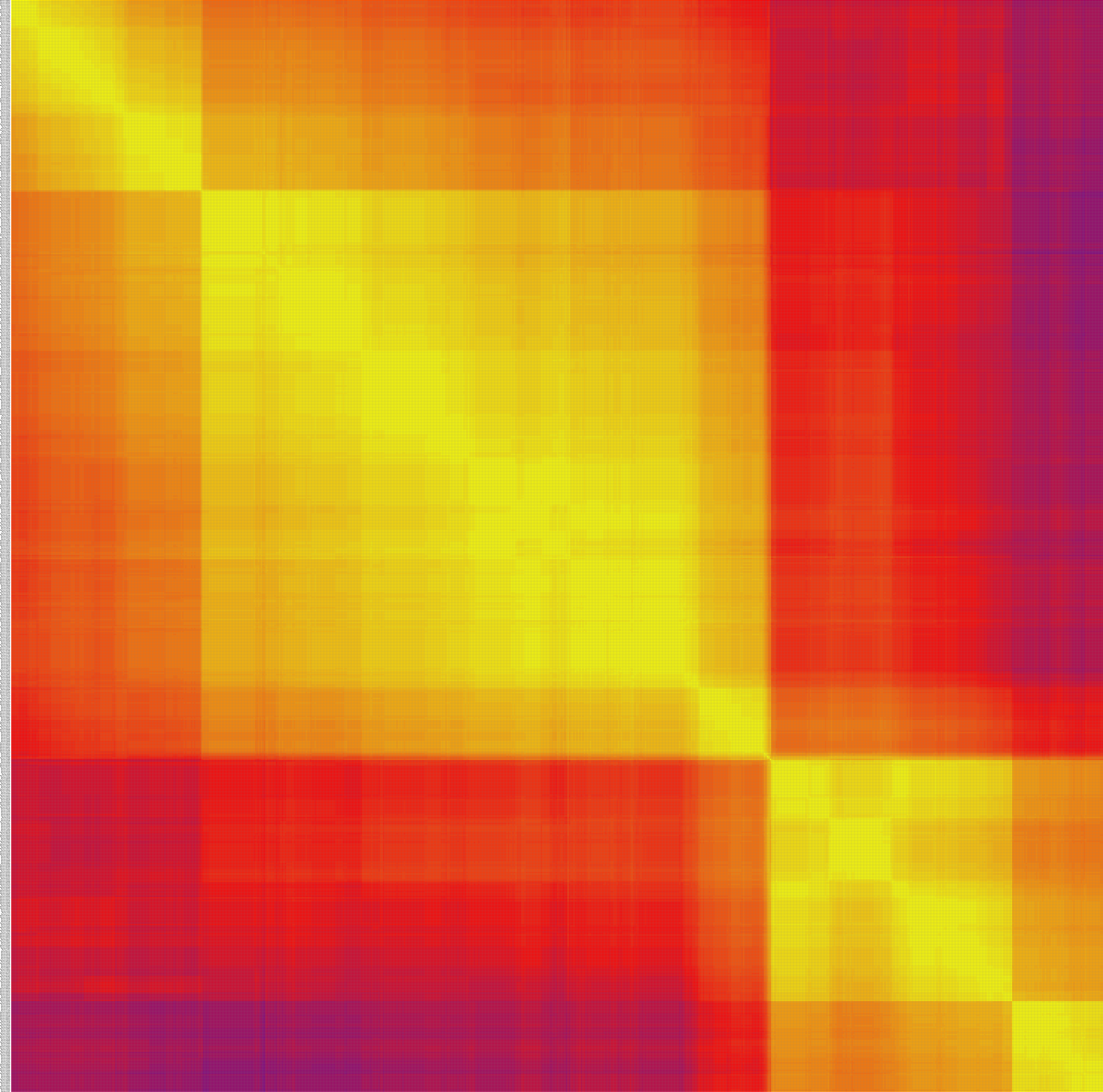

Supplement: FIGURE S1 — Haplotype maps of LG1-LG7. [file Data_Sheet_1.ZIP › Supplemental materials/Supplement Figure/Figure S2/LG3.heatMap.jpg]

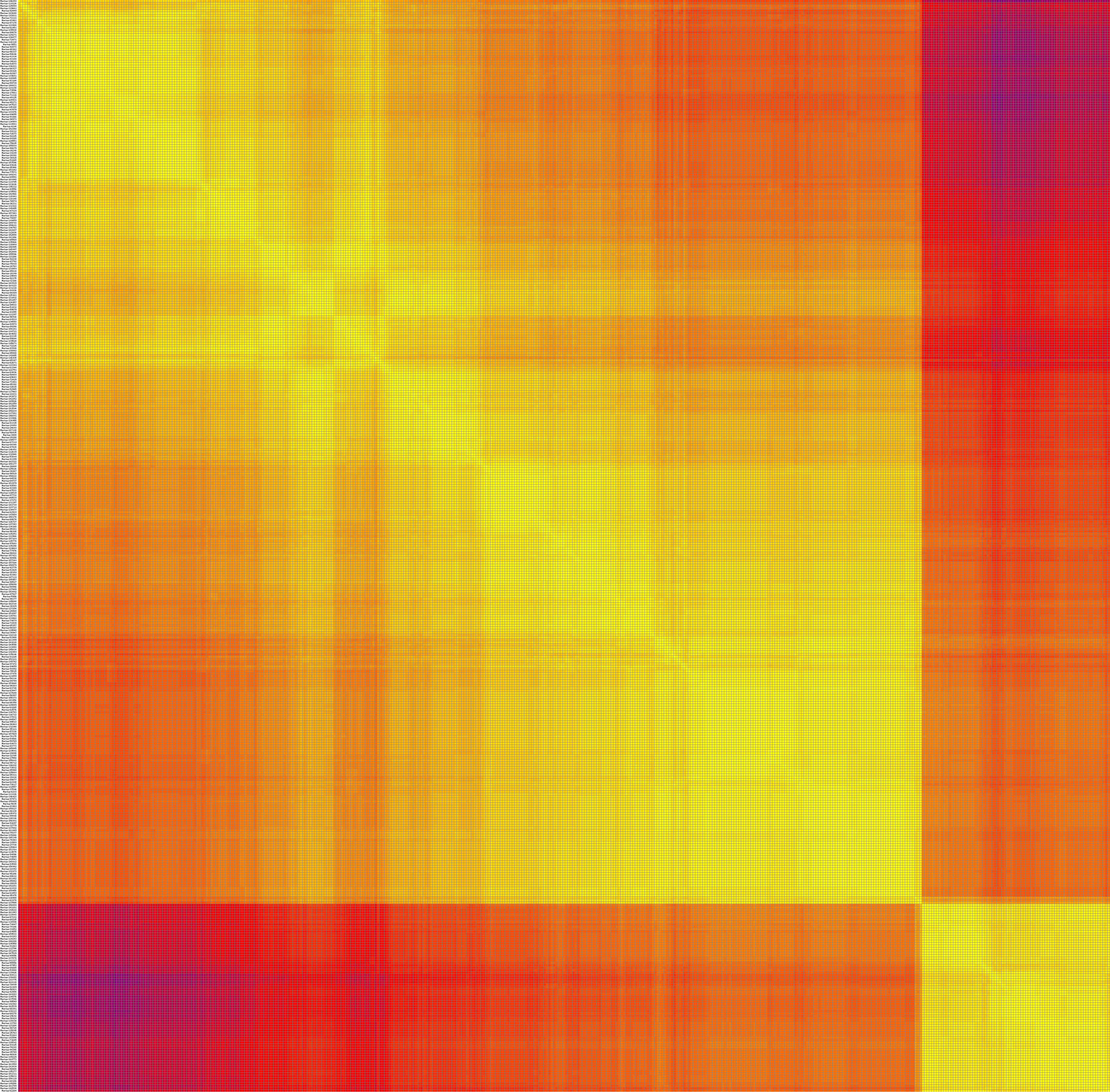

Supplement: FIGURE S1 — Haplotype maps of LG1-LG7. [file Data_Sheet_1.ZIP › Supplemental materials/Supplement Figure/Figure S2/LG4.heatMap.jpg]

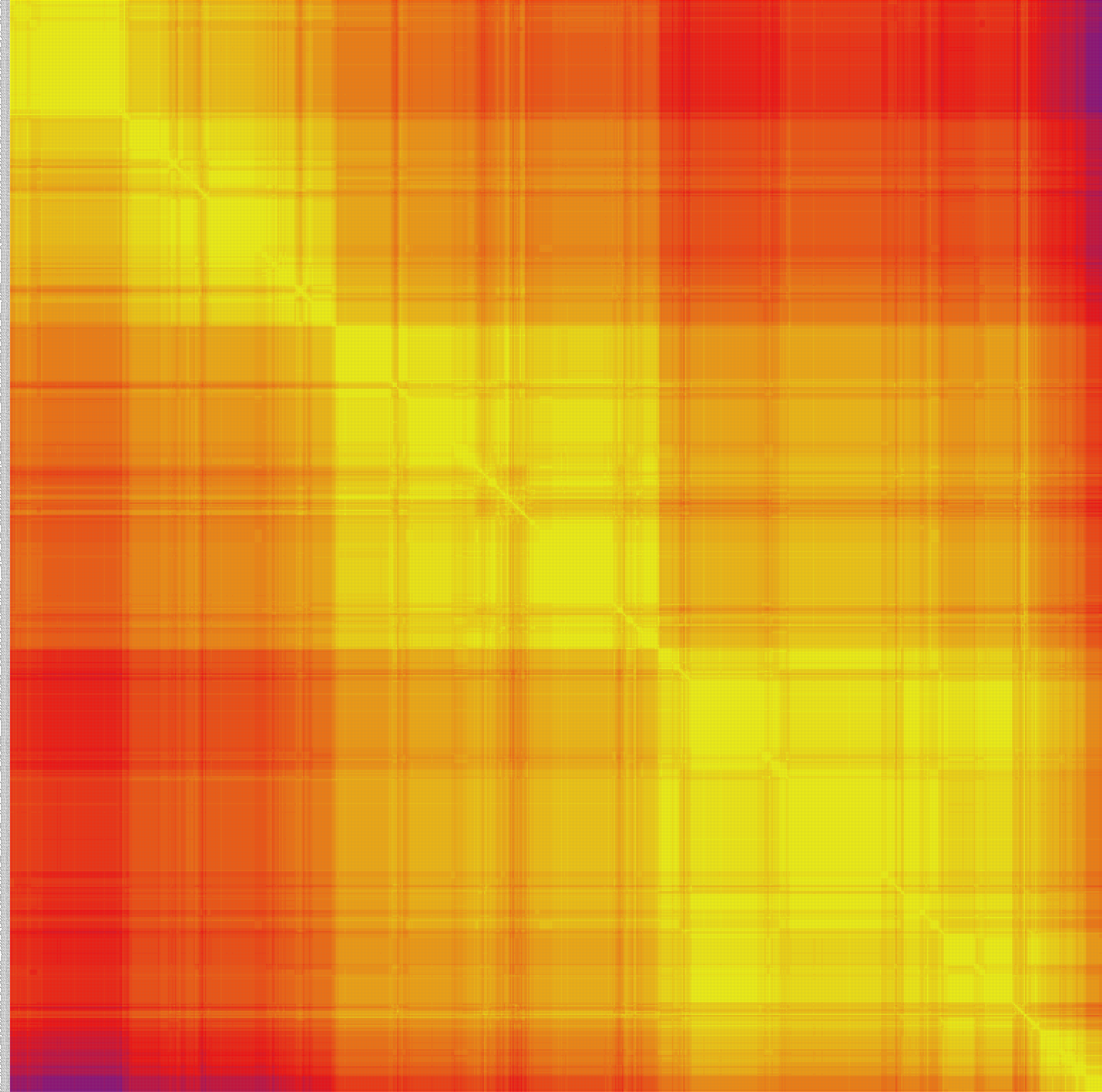

Supplement: FIGURE S1 — Haplotype maps of LG1-LG7. [file Data_Sheet_1.ZIP › Supplemental materials/Supplement Figure/Figure S2/LG5.heatMap.jpg]

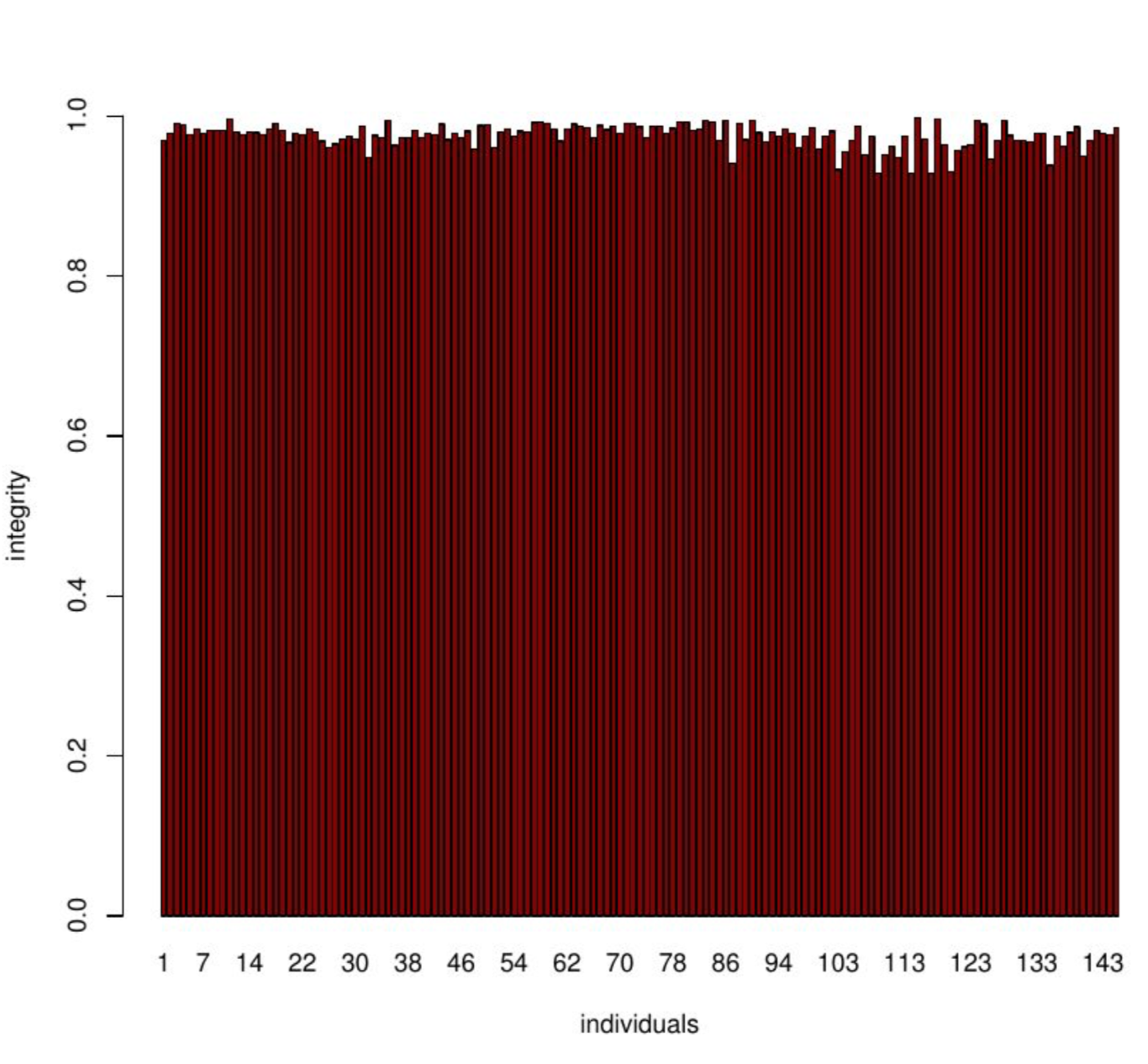

Supplement: FIGURE S1 — Haplotype maps of LG1-LG7. [file Data_Sheet_1.ZIP › Supplemental materials/Supplement Figure/Figure S3.jpg]

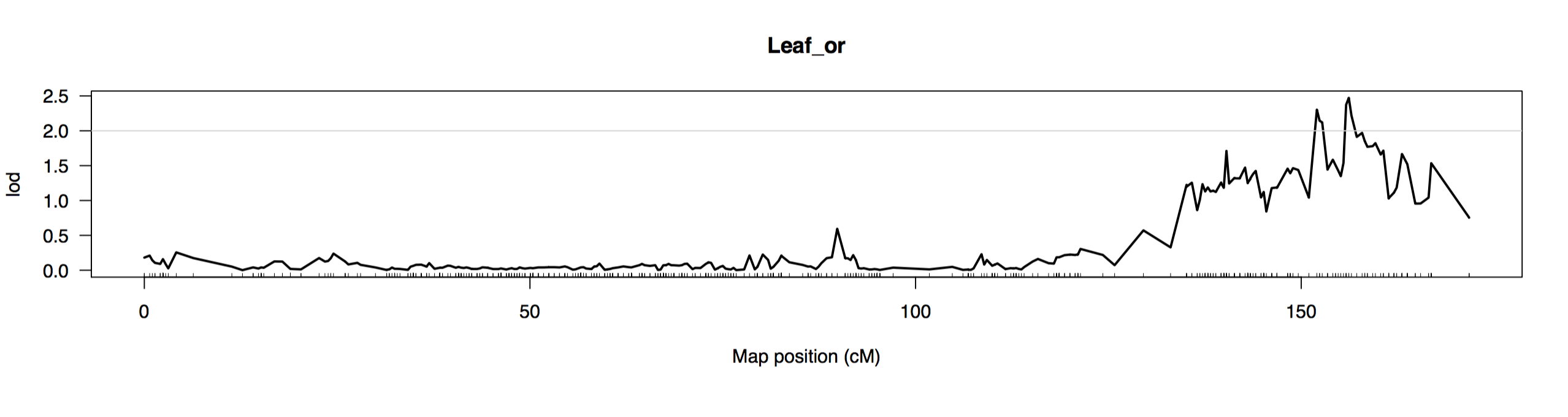

Supplement: FIGURE S1 — Haplotype maps of LG1-LG7. [file Data_Sheet_1.ZIP › Supplemental materials/Supplement Figure/Figure S4.jpg]
